# Distinct Domains within the Human Cytomegalovirus U_L_26 Protein Are Important for Wildtype Viral Replication and Virion Stability

**DOI:** 10.1371/journal.pone.0088101

**Published:** 2014-02-05

**Authors:** Chun Mathers, Cody M. Spencer, Joshua Munger

**Affiliations:** Department of Biochemistry and Biophysics, University of Rochester Medical Center, Rochester, New York, United States of America; National Institute of Allergy and Infectious Diseases, United States of America

## Abstract

The human cytomegalovirus (HCMV) U_L_26 gene encodes a virion protein that is important for high titer viral replication. To identify specific domains within the U_L_26 protein that contribute to viral infection, we created a panel of site-directed U_L_26 mutant viruses and assessed their impact on phenotypes attributed to U_L_26. We find that the C-terminal 38 amino acids of the U_L_26 protein are absolutely necessary for U_L_26 function. A stop-insertion mutant that produced a truncated U_L_26 protein lacking this region behaved identically to U_L_26-null viruses. This included reduced accumulation of IE1 protein at early time points, smaller plaque size, reduced virion stability, and growth with similarly attenuated kinetics. This C-terminal truncation decreased the amount of U_L_26 packaged into the virion resulting in reduced delivery of U_L_26 to newly infected cells. Further, this C-terminal truncated U_L_26 exhibited substantially reduced nuclear localization compared to wildtype U_L_26. Translation of U_L_26 mRNA is initiated from two separate in frame methionines that give rise to a long and a short isoform of U_L_26. We find that the N-terminal 34 amino acids, which are unique to the long isoform of U_L_26, are also important for the function of the U_L_26 protein. A viral mutant that produces only the short isoform of U_L_26 and lacks these N-terminal 34 amino acids exhibits delayed IE1 accumulation, and demonstrates intermediate defects in viral plaque size, virion stability and viral growth kinetics. Ablation of the short U_L_26 isoform in the presence of the long U_L_26 isoform did not impact any of the *in vitro* phenotypes tested. These experiments highlight important domains within the U_L_26 protein that contribute to HCMV infection.

## Introduction

Human cytomegalovirus (HCMV), a betaherpesvirus, is a widespread opportunistic pathogen. HCMV causes severe disease in various immunosuppressed populations including the elderly, cancer patients receiving immunosuppressive chemotherapy, transplant recipients, and AIDS patients [1], [2]. HCMV infection is also a substantial cause of rejection in allograft recipients (kidney, liver, heart and bone marrow) [3]–[6]. Further, congenital HCMV infection is a major cause of birth defects resulting in permanent disabilities in approximately one in a thousand live births [7]–[9]. Congenital HCMV infection can result in multiple organ system abnormalities, although central nervous system damage is the most prevalent sequelae, which occurs in the majority of symptomatic newborns [2], [10].

HCMV is a relatively large virus, with a ∼240-kb DNA genome that encodes >200 open reading frames. The viral particle is enveloped and its genome is encased within a protein capsid. Packaged in between the capsid and the viral envelope is a protein layer called the tegument, a structural feature unique to herpes viruses [11]–[13]. Tegument proteins perform diverse functions during viral infection. Some tegument proteins are important for structure and assembly of virions such as those encoded by U_L_32 and U_L_99 [14]–[16]. Tegument proteins are delivered to the cellular cytoplasm upon viral membrane fusion and many function at the earliest steps of infection. Such examples include pp71, which serves as a transcriptional activator of viral genes, and also suppresses the Rb tumor suppressor [17]–[19], and pp65 which antagonizes innate immunity and the interferon response [20], [21]. Tegument proteins are therefore critical at multiple steps during HCMV infection; at early times, they initiate a cellular environment conducive to viral replication, and later, they help assemble viral particles.

While many HCMV tegument proteins are known to be important for HCMV replication, the mechanisms through which many of these proteins contribute to the infectious cycle are unclear. One such tegument protein is encoded by the U_L_26 gene, which has been found to be critical for high-titer viral replication [22]–[24]. The U_L_26 protein is expressed with early kinetics, and synthesis of the protein initiates at one of two start codons resulting in 21- or 27-kDa products [25]. HCMV strains containing a U_L_26 deletion grow to lower final titers, with slower growth kinetics, and exhibit a small plaque phenotype [23], [24]. U_L_26 has been implicated in transcriptional activation of the immediate early promoter [23], [25]. Deletion of U_L_26 also impacts the structural characteristics of virions. These mutants are less stable than wildtype virions and contain hypophosphorylated tegument constituents [23], [24]. Consistent with a nuclear function early during infection, and a role in viral assembly at late time points, the U_L_26 protein localizes to the nucleus at early times post infection, and to viral assembly compartments at late time points [23].

Here, we analyzed specific domains of U_L_26 that contribute to U_L_26-dependent phenotypes through the creation of a panel of mutant U_L_26 viruses. Site-directed mutagenesis was employed to target both of U_L_26’s initiation methionines and to introduce stop codons throughout the U_L_26 ORF. Analysis of these mutant viruses indicates that the U_L_26 short isoform is dispensable *in vitro* when in the presence of the U_L_26 long isoform. In contrast, the extra N-terminal 34 amino acids of the long U_L_26 isoform was found to be important for U_L_26-dependent phenotypes, exhibiting intermediate defects in plaque size and virion stability in comparison to wildtype and U_L_26-null viruses. Lastly, the carboxy terminal 38 amino acids were found to be critical for wildtype replication as deletion of this domain phenocopied U_L_26-deletion viruses. Deletion of these of 38 amino acids reduced the nuclear localization and tegumentation of the resulting U_L_26 protein product. These studies indicate that distinct domains of U_L_26 contribute to different U_L_26-dependent phenotypes and shed light on how these domains contribute to HCMV replication.

## Materials and Methods

### Cell Culture and Viruses

MRC5 fibroblasts (passages 23–29) were cultured in Dulbecco’s modified Eagle medium (DMEM; Invitrogen) supplemented with 10% fetal bovine serum. The wild type HCMV strain used in this study was *BAdwt*, a bacterial artificial chromosome (BAC) clone of Ad169 [26], [27]. Cells were grown to ∼ 3.2×10^4^ cells per cm^2^. Prior to infection, cells were serum starved for 24 hours. In all infections, viral innocula were added to cells for a 2 hr adsorption period and then aspirated. For experiments involving measurement of viral titers via plaque assay, unbound virus was inactivated through a sodium citrate wash (40 mM sodium citrate, 10 mM KCl, and 135 mM NaCl, pH 3.0) followed by a DMEM wash immediately following viral adsorption.

### BAC Mutagenesis

All U_L_26 mutants were derived from the *BAdwt* clone of Ad169 (Genebank accession number: FJ527563) [26], [27]. The U_L_26 mutants constructed are: BAdUL26 double methionine deletion (referred to as DBmetΔ in the text); BAdUL26 1^st^ Methionine deletion (referred to as 1^st^metΔ in the text); BAdUL26 2^nd^ Methionine deletion (referred to as 2^nd^metΔ in the text); BAdUL26 double methionine deletion rescue (referred to as DBrescue in the text); BAdUL26 #68 stop codon mutant (referred to as #68stop in the text); BAdUL26 #107 stop codon mutant (referred to as #107stop in the text); BAdUL26 #146 stop codon mutant (referred to as #146stop in the text); BAdUL26 #185 stop codon mutant (referred to as #185stop in the text). Wild type B*Adwt* is referred as WT in the text and BAdUL26 transposon insertion virus [22] is referred to as UL26TI in the text. Red recombineering was used to construct the viral mutants in either a one-step or two-step PCR recombination process as previously described [28]. Briefly, for the two step PCR, a PCR amplified Kan/Isce I cassette from the pEPkan-S vector containing U_L_26 flanking sequences was recombined into BAD*wt* through electroporation into *E. coli (*strain SW105) containing *BAdwt*. Recombination was screened by growth in kanamycin. The Kan/Isce I cassette containing BAC was then electroporated into GS1783 cells, which contain an arabinose-inducible I-Sce 1 restriction site used for negative selection [29]. In the second step of Red recombineering, a double-stranded DNA oligo containing the mutant sequence of interest was transformed into GS1783 competent cells containing the Kan/Isce I cassette to allow recombination and insertion of the mutant sequence into the Kan/Isce I cassette site. Recombinants were negatively selected on arabinose, positively selected on chloramphenicol and screened for loss of kanamycin resistance. Restriction enzyme analysis of all BAC clones was performed to rule out large-scale aberrant recombination events. Further, all recombinant BAC clones were sequenced to confirm the presence of the inserted mutation and confirm the lack of any additional mutations in the U_L_26 gene. The UL26 DBmetΔ mutant was created by deletion of the 2^nd^ Methionine from the UL26 1^st^metΔ mutant. The primers for generating mutant viruses via two sequential PCR reactions (first reaction to introduce Kan/Isce I cassette and second reaction to insert the point mutation) were as follows (5′ to 3′): UL26 1stmetΔ insertion:F-GGCCCTCGGTGCGCTACCGGGCCCACATTCAAAAGTTTGAGCGTC

TTCATAGGATGACGACGATAAGTAGGG; R-GCGGCTTCATGTGGCGTGACCT

CCGACCTCGTGAGGCCGAAAACGGCGTACAACCAATTAACCAATTCTGATTAG;

UL26 1^st^metΔ Met to ATC: F -GCGCTACCGGGCCCACATTCAAAAGTTTGAGCGT

CTTCATCTACGCCGTTTTCGGCCTCACGAGGTCGGAGGTCACGCCA;

R–TGGCGTGACCTCCGACCTCGTGAGGCCGAAAACGGCGTAGATGAAGA

CGCTCAAACTTTTGAATGTGGGCCC GGTAGCGC; UL26 1^st^met to ATC negative control:F-GCGCTACCGGGCCCACATTCAAAAGTTTGAGCGTCTTCATGTACGCCGT

TTTCGGCCTCACGAGGTCGGAGGTCACGCCA; R- TGGCGTGACCTCCGACCT

CGTGAGGCCGAAAACGGCGTACATGAAGACGCTCAAACTTTTGAATGTGGGCCCGGTAGCGC;UL26 2^nd^metΔ insertion:F-GCGGCGCGTTATAAGCACCGTGG

GGTCATCGACCGACAAGGCGCGGCGATAGGATGACGACGATAAGTAGGG;R- CGCATAAAATCGTCTAAATTCAAACCGCCGTCGGGTGCGCGCCTACTCGTCAACCAATTAACCAATTCTGATTAG; UL26 2^nd^metΔ Met to ATC: F-ATAAGCACCGTGGGGT

CATCGACCGACAAGGCGCGGCGATCACGAGTAGGCGCGCACCCGACGGCGGTTTGAATTTAGAC;R-GTCTAAATTCAAACCGCCGTCGGGTGCGCGCCTACTCGTGAT

CGCCGCGCCTTGTCGGTCGATGACCCCACGGTGCTTAT; UL26 2^nd^met to ATC negative control:F-ATAAGCACCGTGGGGTCATCGACCGACAAGGCGCGGCGATGA

CGAGTAGGCGCGCACCCGACGGCGGTTTGAATTTAGAC;R-GTCTAAATTCAAAC

CGCCGTCGGGTGCGCGCCTACTCGTCATCGCCGCGCCTTGTCGGTCGATGACCCCACGGTGCTTAT; UL26 DBmetΔ: F-CGCCGTTTTCGGCCTCACGAG; R-GGTGCC

GATGACGCGCAACTG. The single step PCR process employs only a single set of primers that contain homology sufficient for both recombination events [28]. Primers used for mutants created through a single PCR reaction are listed: UL26#185stop:F-CACGGTGACGTAGCAGCACGCGGCTCACGTAGCAGGCCGATTAGCGGATGACCTGGCCGTCGGAGGATGACGACGATAAGTAGGG;R-CTCGGGCCTGCGACG

CGACGCCGACGGCCAGGTCATCCGCTAATCGGCCTGCTACGTGAGCCGCA

ACCAATTAACCAATTCTGATTAG; UL26#146stop:F-GCTCCACGTCTTCAA

AGTAGCTGTGTAGCAGGCCGCGCTCTTACAGCTGCGGCAGCGAGT

CGGAGGATGACGACGATAAGTAGGG; R-GAACTTTGTAGTGCGCGCC

GCCGACTCGCTGCCGCAGCTGTAAGAGCGCGGCCTGCTACACAGCAACCAATTAACCAATTCTGATTAG; UL26#107stop:F –CGGCCGCCACGCCGGCCACGCTGC

GGTCCCAACTGAAAAGTTAGGCGAGTCCGATGGTGCCGAAGGATGACGACGATAAGTAGGG; R-CCAGGGTCAGTTGCGCGTCATCGGCACCATCGGACTCGCCT

AACTTTTCAGTTGGGACCGCAGCAACCAATTAACCAATTCTGATTAG; UL26#68stop:F-GCGGGGTGAGGATGGTCTCCTCCACGTCGCAGACAAACAATTA

GTAGCCGCGCGGATAGGGCAAGGATGACGACGATAAGTAGGG; R-GCGCGGT

CGCCACCTGGATCTGCCCTATCCGCGCGGCTACTAATTGTTTGTCTGCGACGTGGACAACCAATTAACCAATTCTGATTAG

### Protein Analysis

Protein accumulation was assayed by Western blotting. Protein from cell lysates was solubilized in disruption buffer (50 mM Tris [pH 7.0], 2% SDS, 5% 2-mercapoethanol, and 2.75% sucrose), separated by either 10% or 15% SDS-PAGE, and transferred to nitrocellulose in Tris-glycine transfer buffer. Blots were then stained with Ponceau S to visualize protein bands and ensure equal protein loading. The membranes were blocked in 5% milk in Tris-buffered saline-Tween 20 (TBST), followed by incubation in primary antibody. After subsequent washes, blots were treated with secondary antibody and protein bands were visualized using the enhanced chemiluminescence (ECL) system (Pierce). The primary antibodies were specific for viral proteins UL123-coded IE1 [1B12], U_L_26 [7H19] (a C-terminal specific antibody), PP28 [10B4–29], UL83-coded pp65 (8F5) and pUL44 [10D8; Virusys] and cellular protein tubulin [Epitomics]. A rabbit UL26 N-terminal specific antibody was generated by Biomatik (http://www.biomatik.com/) using the following underlined sequence: MTSRRAPDGGLNLDD. The methionine preceding this sequence is the 2^nd^ U_L_26 initiation methionine. The secondary antibodies were rabbit polyclonal [Santa Cruz Biotechnology, Inc.] and mouse monoclonal [Abcam].

**Viral DNA accumulation** was monitored by real-time PCR. At various times post infection, medium was aspirated from cells and viral DNA was harvested in lysis buffer (100 mM NaCl, 100 mM Tris-HCl, 25 mM EDTA, 0.5% SDS, 0.1 mg/ml proteinase K, and 40 µg/ml RNase A). The extracted nucleic acid was quantified and checked for purity through 260∶280 absorbance by NanoDrop. Quantitative PCR (qPCR) was performed using Fast SYBR green master mix, a model 7500 Fast real-time PCR system, and Fast 7500 software (Applied Biosystems) according to manufacturer’s instructions. Viral DNA was quantified with specific primer pairs targeting U_L_83 (pp65), 5′-CAG-GAA-GAT-TTG-CTG-CCC-GTT-CAT-3′ (forward) and (5′-GGC-TTT-ACG-GTG-TTG-TGT-CCC-AAA-3′ (reverse).

**For immunofluorescence**, MRC5 fibroblasts were grown on glass coverslips. At various time points post infection, cells were washed once with PBS, fixed with 2% paraformaldehyde in PBS for 20 min, washed three times with PBS, and permeabilized with 0.1% Triton X-100 and 0.1% SDS for 15 min, then washed twice with PBS containing 0.05% Tween-20. Cells were subsequently blocked by overnight incubation in PBS containing 2% bovine serum albumin (BSA), 5% goat serum, 5% human serum, and 0.3% Triton X-100. Cells were incubated with anti-U_L_26 sera [7H19] that had been diluted 1∶2 in PBS containing 0.05% Tween-20 for 1 hr. Slides were subsequently washed with PBS containing 0.01% Tween-20 three times, incubated with fluorochrome-conjugated anti-mouse secondary antibody for 1 hr, and washed three times in the same buffer lacking antibody. Coverslips were mounted in slow-fade Gold antifade reagent (Molecular Probes) and DAPI (4′, 6′-diamidino-2-phenylindole). Confocal images were captured with FV1000 Olympus laser scanning confocal microscope. All images were captured under identical confocal settings.

### Virion Purification

To produce partially purified virions for the analysis of their constituent proteins, WT and #185stop virus stocks were first clarified by low speed centrifugation and then centrifuged through a sorbitol cushion at 26K rpm for 1 hr. The virion pellet was then resuspended in T.N. buffer containing 20 mM Tris-HCl, PH 7.4, 100 mM NaCl, and 1.5% BSA and purified by centrifugation through a glycerol tartrate gradient as previously described [23]. Bands containing virions were collected and diluted 4-fold with T.N. buffer. Virions were repelleted by centrifugation at 21 K rpm for 1 hr and resuspended in T.N. buffer. For western analysis of viral preps, disruption buffer was added to a final concentration of 50 mM Tris [pH 7.0], 2% SDS, 5% 2-mercapoethanol, and 2.75% sucrose prior to loading on SDS page gels. Serial dilutions of viral preps were analyzed by anti-U_L_26 and anti-pp65-specific western to ensure the linearity of U_L_26 and pp65 detection. Quantitation of U_L_26 and pp65-specific protein bands was performed using ImageLab software tools from BioRad.

### Analysis of Viral Plaque Formation and Viral Stability

Replicate cultures of MRC5 fibroblasts were infected with 25 PFU of the indicated recombinant virus. Representative plaques at day 15 post infection for each virus are shown. Areas of representative plaques for each virus were quantified by Image J and normalized to the WT plaque size. To investigate the stability of virion infectivity, an equivalent number of plaque forming units from freshly thawed viral stocks were incubated at 37°C for 0, 4, 8, or 20 hours. After the indicated incubation period, confluent MRC5 fibroblasts were infected. The percentage of plaques remaining relative to the 0 h control was plotted.

To investigate virion stability after trypsin exposure, infected MRC5 cells (MOI = 3.0) were harvested when the CPE reached 80%. The media containing infectious virus was reserved, and cells were scraped in a small volume of media and sonicated. The sonicated cells and reserved culture media were combined and centrifuged at 6,000 rpm for 30 min. The supernatants were then sedimented at 38,000×g for 60 min. The pellets containing virus were resuspended in serum-free minimal essential medium. Two hundred µl of either 2.5% trypsin (Invitrogen) or media was mixed with 1.8 ml of the resuspended virus, and incubated at 37°C for 30 or 60 minutes for the trypsin treated, or 0 minutes for the media control. To inactivate the trypsin, at the end of the prescribed intervals, calf serum was added to a final concentration of 10%. The suspension was then tittered by plaque assay. The percentage of plaques remaining relative to the media control was plotted.

### Statistical Analysis

Statistical significance was assessed by a non-paired two tailed homoscedastic student’s *t*-test unless otherwise indicated. A probability of value (p) <0.05 was considered statistically significant. For comparison of the viral growth between wildtype and the #185stop mutant from 48–120 hpi a homoscedastic paired two-tailed ttest of viral titers at each time point was performed. Averages are plotted with either standard deviation (SD), or standard error of the mean (SE) as indicated.

## Results

### Construction of U_L_26 Mutant Viruses

**Figure 1:**
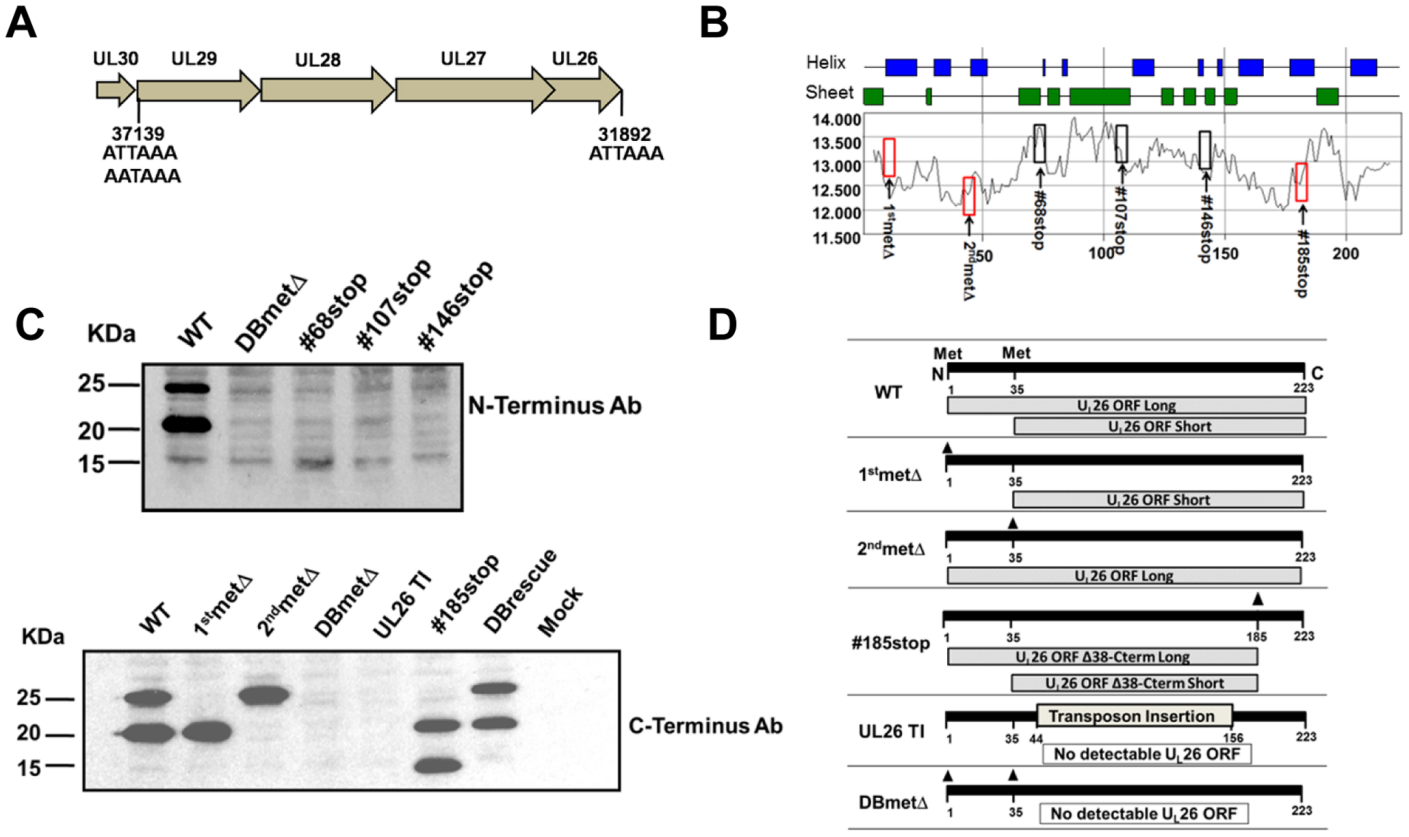
Creation of U_L_26 mutant viruses. (**A**) Schematic of the HCMV U_L_30-U_L_26 genomic region. (**B**) A Manavalan hydrophobicity plot of the U_L_26 protein with the positions of specific methionine mutations or stop insertions illustrated. In blue and green, are Chou-Fassman predicted secondary structure domains. BAC-engineered stop and start mutations are overlaid, with red boxes indicating stable U_L_26 protein accumulation, and black boxes indicating lack of stable U_L_26 protein accumulation. (**C**) Accumulation of viral proteins after HCMV infection. Replicate cultures of fibroblasts were mock infected or infected with WT, 1^st^metΔ, 2^nd^metΔ, DBmetΔ, UL26TI, DBrescue or stop codon mutants: #68stop, #107stop, #146stop and #185stop virus (MOI = 3). Cells were harvested at 48 hr post infection and processed for Western blotting using a U_L_26-specific monoclonal antibody (targeting the N-terminus or C-terminus of U_L_26 as indicated). (**D**) Illustration of the panel of U_L_26 mutant viruses analyzed with the resulting U_L_26 open reading frames (ORFs) indicated.

Viruses containing deletions in the U_L_26 gene have been shown to be growth attenuated [23], [24]. These viruses grow to reduced titers, have decreased virion stability and exhibit decreased plaque size [23], [24]. The U_L_26 protein is expressed from a spliced mRNA transcript that is also responsible for the expression of the U_L_29, U_L_28, U_L_27, and U_L_29/28 open reading frames [25], [30] (Fig. 1A). Previously characterized U_L_26-null viruses contain large deletions within the U_L_26 open reading frame which could impact the expression of the other open reading frames that are expressed from this mRNA transcript. Further, it is unclear how the two separate isoforms of the U_L_26 protein contribute to HCMV infection. To address these issues, and to map the domains of U_L_26 that impact viral replication, we employed BAC-mediated recombineering to create a panel of viruses containing site-directed U_L_26 mutations. This panel included viruses containing a mutation ablating one or both initiating methionines as well as viruses containing stop codon insertions throughout the U_L_26 open frame.

A plot of manavalan hydrophobicity [31] and Chou-Fasman predicted secondary structure domains [32] of the U_L_26 protein is illustrated in Figure 1B. The positions of specific methionine mutations or stop insertions that were engineered into BAC-Ad169 (WT) are illustrated on this plot (Fig. 1B). In an attempt to create truncated U_L_26 reading frames that could be stably expressed, stop insertions were made at locations that approximated transitions between these predicted domains (Fig. 1B). To verify expression of these C-terminally-truncated U_L_26 open reading frames, a rabbit polyclonal antibody was raised to a peptide in the N-terminus of U_L_26. As shown in Figure 1C, three stop insertion viruses did not accumulate any truncated U_L_26 protein whereas one stop insertion mutant, #185stop, did accumulate truncated U_L_26. Mutagenesis of U_L_26’s two initiation methionines gave the expected results; a virus containing a mutation in the first methionine expressed only the short U_L_26 isoform, whereas a virus with a mutation of the second methionine expressed only the long U_L_26 isoform (Fig. 1C). Further, the virus with both methionines mutated did not accumulate any U_L_26 (DBmetΔ), nor did the previously described transposon-deleted U_L_26 virus (UL26TI) (Fig. 1C). The repair of the DBmetΔ virus, through recombination with a DNA fragment containing the wildtype U_L_26 N-terminus created a virus, DBrescue, which restored the expression of both U_L_26 isoforms (Fig. 1C). The stop codon insertion mutants that did not accumulate detectable amounts of U_L_26 behaved like U_L_26-null viruses (data not shown) and were not analyzed in further detail. The specific mutations of the remaining recombinant viruses, and the U_L_26 ORFs they produce, are illustrated in Fig. 1D.

### N-terminal and C- terminal Domains of the U_L_26 Protein are Necessary for Wild-type Replication

**Figure 2:**
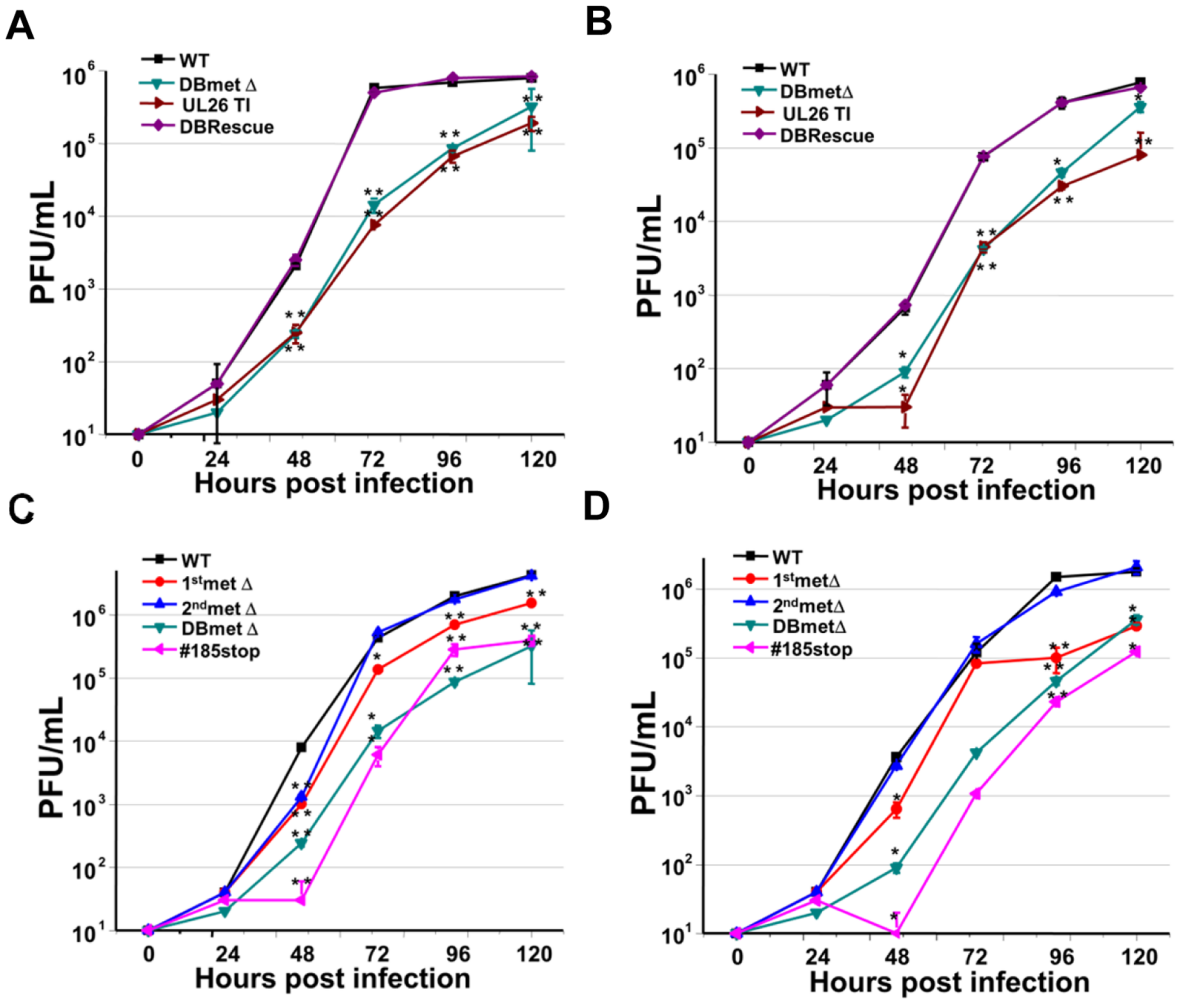
Growth characteristics of U_L_26 recombinant viruses. Serum starved MRC5 fibroblasts were infected with WT, UL26TI, DBmetΔ or DBrescue viruses at an MOI of 3.0 (**A**) or 0.25 (**B**) in triplicate. For **C** **&** **D**, cells were infected with WT, 1^st^metΔ, 2^nd^met Δ, DBmetΔ or #185stop at an MOI of either 3.0 (**C**) or 0.25 (**D**). Supernatants were harvested at the indicated times, and infectious viral progeny was quantified by plaque assay on fibroblasts. Values are means ± SE (n ≥3), and statistical significance was assessed through comparison of mutant to WT titer at the same time post infection by student’s ttest, * = p<0.05; ** = p<0.01.

As it has previously been found that deletion of the U_L_26 protein impacts production of viral progeny, we wanted to elucidate how specific domains of the U_L_26 protein contribute to viral replication. We first wanted to assess whether the double methionine deletion mutant (DBmetΔ), grew with similar kinetics as the transposon insertion mutant (UL26TI). As shown in Figure 2A and 2B, the DBmetΔ virus grew with similar kinetics at both high (3.0) and low (0.25) multiplicities of infection (MOI). Further, repair of the double methionine mutation (DBrescue) restored the viral growth kinetics to wildtype levels (Fig. 2A and 2B). These results indicate that the transposon insertion and subsequent large deletion of the U_L_26 ORF does not substantially impact the *in vitro* viral growth over and above what is observed with less disruptive targeting of the UL26 initiation methionines.

To further determine how different domains of the U_L_26 protein contribute to viral growth, we analyzed the replication of the individual methionine mutants and the #185 stop insertion mutant. At high MOI, the 2^nd^metΔ virus, which only expresses the long isoform of U_L_26 (Fig. 1C), grew similarly to WT (Fig. 2C). The 1^st^metΔ virus, which expresses only the short isoform of U_L_26 (Fig. 1C), displayed an intermediate growth defect. Compared to WT virus, it grew with slower kinetics and exhibited a 5-fold decrease in final titers, but grew better than a virus with the complete U_L_26 deletion (DBmetΔ) (Fig. 2C). The #185stop mutant was more substantially attenuated, growing with reduced kinetics and to lower final titers, similar to the DBmetΔ mutant. Compared to WT virus, the stop mutant exhibited a ∼10-fold reduction in final titers (Fig. 2C). Similar trends were observed during infection at a lower MOI. The 2^nd^metΔ virus grew almost identically to WT, whereas the 1^st^metΔ virus displayed WT growth kinetics early during infection, but exhibited a 10-fold reduction in viral titers (Fig. 2D). Interestingly, the #185stop mutant actually grew statistically worse than the DBmetΔ mutant based on a paired two-tailed students ttest of viral titers from 48–120 hpi (p<0.05) (Fig. 2D). The combined analyses of viral growth suggest that the short U_L_26 isoform is dispensable for HCMV growth *in vitro*. The additional N-terminal 34 amino acids present in the long but absent in the short U_L_26 isoform were found to be important for wildtype levels of *in vitro* viral growth. The short isoform still contributes to viral growth inasmuch as it grows better than the U_L_26-null virus. In contrast, the C-terminal 38 amino acids of the U_L_26 appear to be essential for U_L_26 function.

### The Impact of U_L_26 Mutations on Viral Protein and Viral DNA Accumulation

**Figure 3:**
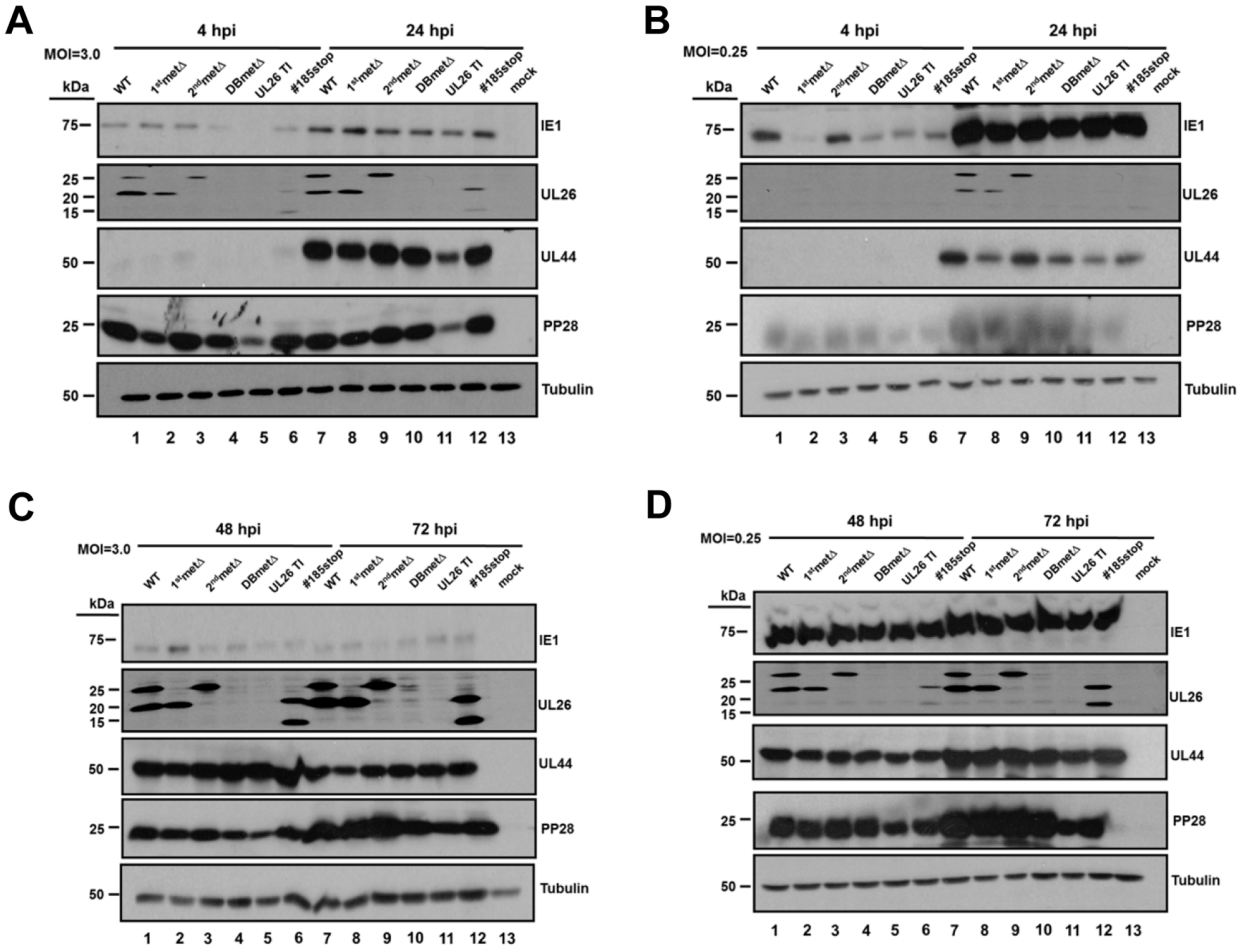
Accumulation of viral proteins after infection with U_L_26 recombinant viruses. Serum starved MRC5 fibroblasts were mock infected or infected with the indicated recombinant virus at an MOI of either 3.0 (**A, C**) or 0.25 (**B, D**). Viral proteins were harvested at 4 hr, 24 hr (**A, B**), 48 hr, 72 hr (**C, D**) post infection and processed for Western blotting using antibodies directed towards IE1, pUL44, pp28, U_L_26 (C-terminal specific antibody), and α-tubulin. A representative blot from two separate experiments is shown.

It has been reported that U_L_26 is important for wildtype levels of IE1 accumulation [23]. To further explore the impact of specific U_L_26 domains on viral gene expression, we analyzed the accumulation of viral proteins during infection. Upon infection at an MOI = 3.0, both U_L_26-null viruses, U_L_26TI and DBmetΔ, accumulated less IE1 at 4 hpi, but recovered by 24 hpi (Fig. 3A). Cells infected with the #185stop mutant appeared to accumulate slightly less IE1 at 4 hpi than WT-infected cells whereas the methionine mutants accumulated WT-levels of IE1 at 4 hpi (Fig. 3A). The impact of U_L_26 mutations on the early accumulation of IE1 was more evident during low MOI infections. At MOI = 0.25, similar to the U_L_26-null viruses, the 1^st^metΔ mutant as well as the #185stop mutant accumulated less IE1 at 4 hpi in comparison to WT infected cells (Fig. 3B). The 2^nd^metΔ mutant, which expresses only the long U_L_26 isoform, accumulated a WT-level of IE1 at 4 hpi (Fig. 3B). As with the high MOI infection, the levels of IE1 protein recovered by 24 hpi (Fig. 3B). Our results indicate that the U_L_26 protein is important for the early accumulation of the IE1 protein. Further, it appears that the N-terminal 34 amino acids and C-terminal 38 amino acids play a role in this phenotype in an MOI-dependent manner.

Transposon-mediated deletion of the U_L_26 protein also resulted in decreased delivery of the tegument protein pp28 upon initial infection [23]. Consistent with the previous observations, cells infected with U_L_26TI contained less pp28 protein at 4 and 24 hpi (Fig. 3A). In contrast, cells infected with the DBmetΔ mutant contained WT levels of pp28 at 4 and 24 hpi (Fig. 3A). This indicates the possibility that transposon-mediated deletion of U_L_26 could have additional consequences separate from the ablation of U_L_26 expression, e.g. a second-site mutation. However, any potential second mutation had negligible impact on infection as repair of the U_L_26 mutation in the transposon mutant rescued viral growth [23]. The accumulation of U_L_26 isoforms was as expected; the 1^st^metΔ and 2^nd^metΔ mutants accumulated only the short or long isoforms respectively whereas neither the DBmetΔ nor UL26TI accumulated U_L_26 (Fig. 3A). During the early stages of infection with the #185stop mutant, there was a reduction in the amount of U_L_26 compared to WT (Fig. 3A). However, by 48 hpi, the accumulation of #185stop-U_L_26 was equivalent to that of WT-U_L_26 (Fig. 3C). Analysis of the accumulation of another early protein, U_L_44, indicated little difference in U_L_44 levels between the panel of viruses at an MOI = 3.0 (Fig. 3A). However, during infection at an MOI = 0.25, the 1^st^metΔ and U_L_26-null viruses accumulated less U_L_44 at 24 hpi compared to WT (Fig. 3B). This difference largely disappeared by 48 hpi (Fig. 3D). In cells infected with the U_L_26-null viruses, there also appeared to be a moderate decrease in the amount of pp28 at 48 and 72 hpi (Fig. 3C, Fig. 3D). This decrease was observed at both high and low MOI infections, and would be consistent with the delayed kinetics of infection observed with U_L_26-null viruses. Taken together, our data indicate that at lower multiplicities of infection, the N-terminal 34 and C-terminal 38 amino acids of U_L_26 are important for the early timing of HCMV viral gene expression, inasmuch as the deletion of these domains results in slower accumulation of IE1, and subsequently U_L_44.

**Figure 4:**
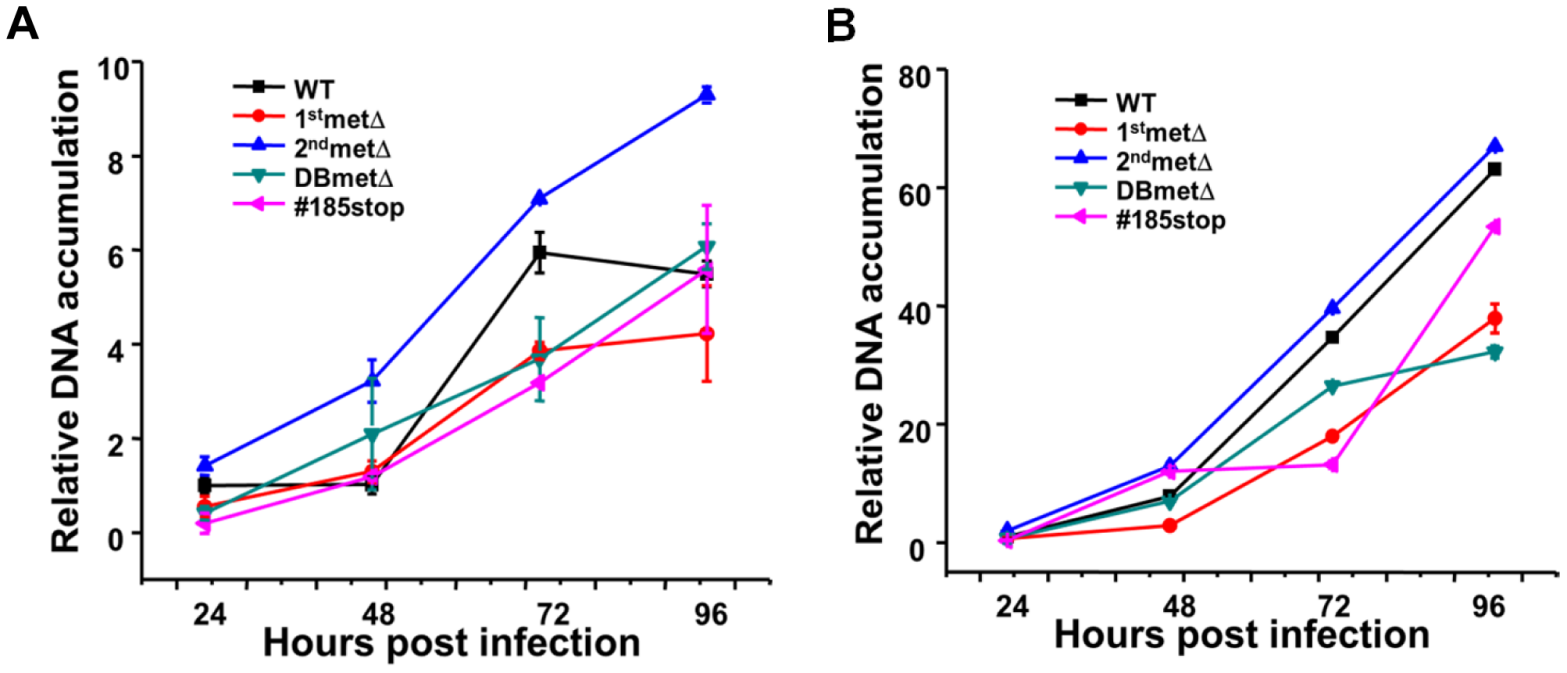
Accumulation of viral DNA after infection with U_L_26 recombinant viruses. (**A**) Serum starved MRC5 fibroblasts were infected with the indicated recombinant virus at an MOI of either 3.0 (**A**) or 0.25 (**B**). Viral DNA was collected at 24, 48, 72 and 96 hpi. Real-time PCR was performed using HCMV-specific primers to analyze viral DNA accumulation. Values are means ± SD (n = 3).

To further analyze the contribution of these U_L_26 domains to the viral infectious cycle, we measured the accumulation of viral DNA over the course of infection with our panel of U_L_26 mutants. At a relatively high MOI (3.0), viral DNA accumulated similarly between the WT and 2^nd^metΔ (Fig. 4A). During infection with the 1^st^metΔ, #185-stop and DBmetΔ viruses viral DNA accumulated with slower kinetics (Fig. 4A). A similar trend was observed at a lower MOI (0.25) single round of infection. Cells infected with the 1^st^metΔ, #185stop and DBmetΔ viruses accumulated DNA less rapidly than cells infected with WT or the 2^nd^metΔ virus (Fig. 4B). These results are consistent with the notion that the N-terminal and C-terminal domains of U_L_26 are important for normal timing of HCMV infection. Deletion of these domains results in a slower accumulation of viral DNA and viral proteins.

### The Impact of U_L_26 Mutations on Viral Plaque Size and Virion Stability

**Figure 5:**
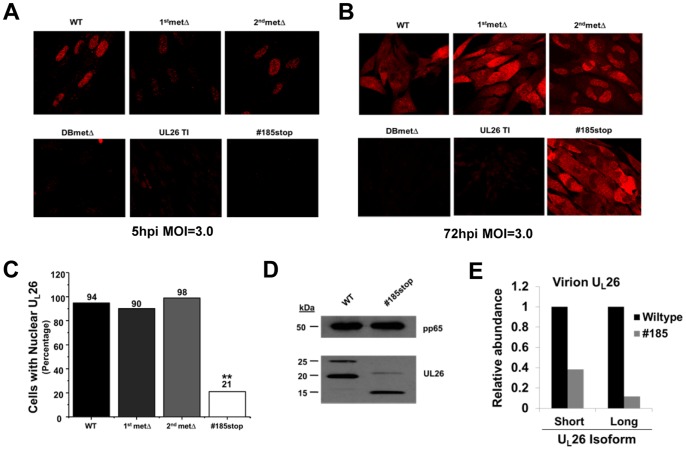
Analysis of HCMV plaque formation. (**A**) Replicate cultures of MRC5 fibroblasts were infected with 25 PFU of the indicated recombinant virus. Representative plaques at day 15 post infection for each virus are shown. (**B**) Areas of representative plaques for each virus were quantified by Image J and normalized to the WT plaque size. Values are means+SE (n = 10). ** = p<0.01; *** = p<0.001.

We and others have observed that deletion of the U_L_26 open reading frame results in reduced plaque size [24]. To analyze the contribution of specific U_L_26 domains to this phenotype, cells were seeded with a fixed number of plaque forming units (PFU) from our panel of mutants, overlaid with agarose and incubated at 37°C for 15 days. Images of the resulting plaques were captured with subsequent analysis of the area of each plaque. Images of representative plaques are shown in Figure 5A. As shown in Figure 5, the resulting plaques were substantially smaller upon infection with the #185 stop or U_L_26-null viruses. The plaques produced by the 1^st^metΔ virus were intermediate in size between the U_L_26-null viruses and WT HCMV, whereas the 2^nd^metΔ virus produced plaques of WT size. These results indicate that the C-terminal 38 amino acids of the U_L_26 protein, and to a lesser extent, the N-terminal 34 amino acids are important for WT plaque size.

**Figure 6:**
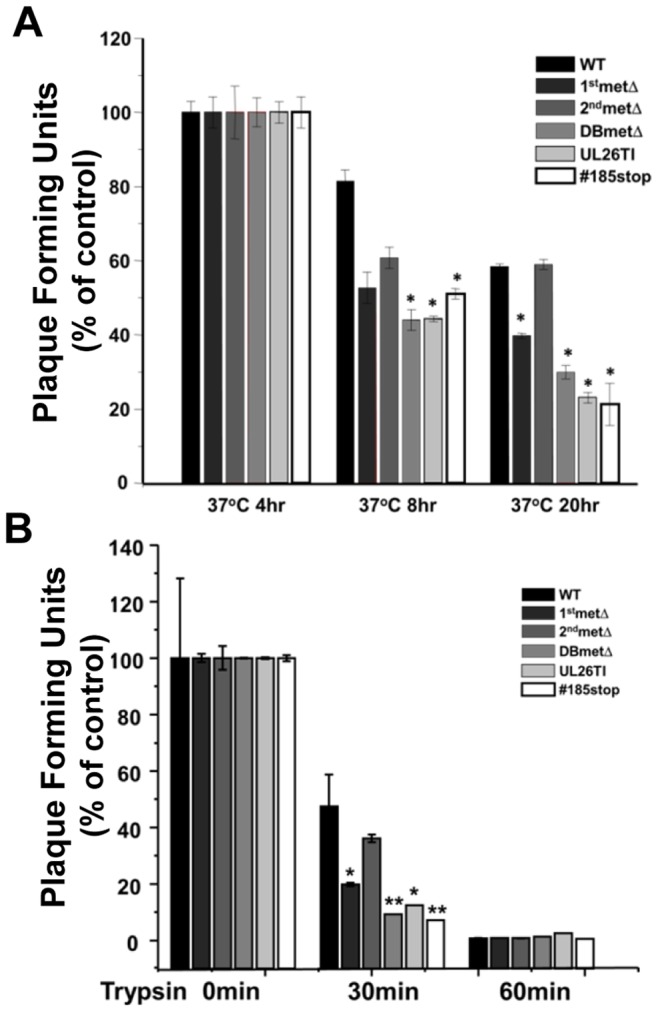
Viral stability of U_L_26 recombinant viruses. (**A**) Replicate cultures of MRC5 fibroblasts were seeded with 25 plaque forming units of WT, 1^st^met Δ, 2^nd^met Δ, DBmetΔ, UL26TI, or #185stop virus that had been incubated at 37°C for 0, 4, 8, or 20 h. The percentage of plaques remaining relative to the 0 h incubation was plotted (e.g. (PFU at T = 0 h incubation)/(PFU at T = 8 h incubation)×100). Values are averages+SE. Statistical significance was assessed through comparison of the decrease in mutant plaquing efficiency relative to WT after the same incubation time, e.g. 8 h at 37°C, by student’s ttest * = p<0.05 (n = 3). (**B**) Replicate cultures of MRC5 fibroblasts were infected with WT, 1^st^met Δ, 2^nd^met Δ, DBmetΔ, UL26TI, or #185stop virus at MOI = 3.0. When the infected cells reached 80% cytopathic effect (CPE), the cells were harvested, and virions partial purified, with subsequent trypsin or control treatment for various times at 37°C. The percentage of plaques remaining relative to non-trypsin treated samples (0 min) was plotted. Values are averages ± SD. * = p<0.05; ** = p<0.01.

Previously, it has been found that the U_L_26 protein is important for virion stability, inasmuch as prolonged incubation at 20°C causes U_L_26-defective virions to lose their ability to initiate infection faster than WT virions do. [24]. To investigate how specific U_L_26 mutations impact virion stability, viral supernatants containing equivalent PFUs were incubated at 37°C for various times and then plated. The percentage of plaques remaining after incubation in comparison to control was plotted in Figure 6A. After 8 h of incubation, WT HCMV exhibited a less than 20% drop in infectivity (Fig. 6A). With the same incubation, the #185stop and U_L_26-null viruses demonstrated a ∼50% drop in infectivity, a statistically significant difference (Fig. 6A). After 20 h of incubation, WT HCMV lost ∼40% of its infectivity (Fig. 6A). With this incubation, the #185stop and U_L_26-null viruses demonstrated a 70% loss of infectivity while the 1stmetΔ lost 60% of its infectivity (Fig. 6A). Different strains of HCMV have historically been found to exhibit varying sensitivities to trypsin treatment, a correlate of virion stability [33]. We therefore explored whether U_L_26 impacts viral trypsin sensitivity. We found that the U_L_26-null viruses, as well as the #185stop and 1stmetΔ viruses exhibited enhanced sensitivity to trypsin compared to wildtype HCMV (Fig. 6B). The infectious stability and trypsin sensitivity results mirror each other, and suggest that the C-terminus of U_L_26, and to a lesser extent U_L_26’s N-terminal 34 amino acids, are important for infectious virion stability.

### The Impact of N-terminal and C-terminal Mutations on U_L_26 Protein Localization

**Figure 7:**
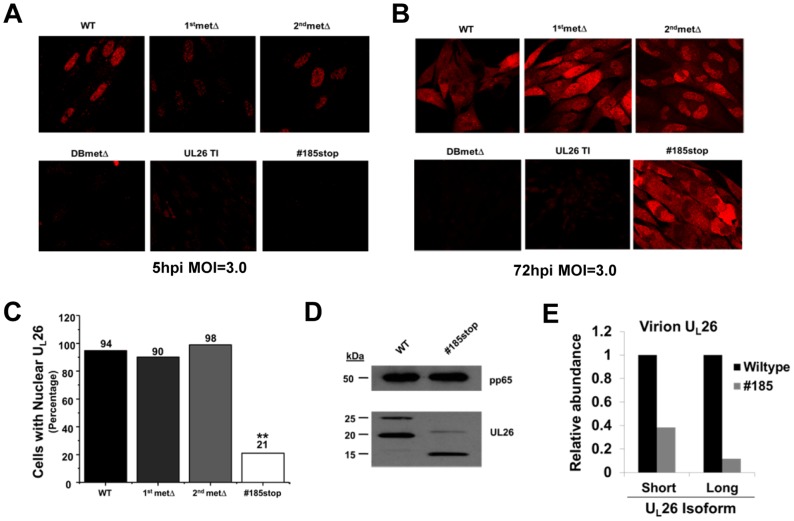
Localization of the U_L_26 protein in HCMV infected cells. MRC5 fibroblasts were infected at an MOI of 3.0 and were fixed at either 5(**A**) or 72 hpi (**B**) and processed for immunofluorescence using an antibody specific for the C-terminus of U_L_26. (**C**) Quantification of the cells containing nuclear localized U_L_26 protein at 72 hpi. The percentage of total cells containing nuclear localized U_L_26 was plotted. U_L_26 localization in over one-hundred cells in separate fields was determined, ** p<0.01 compared to WT virus. All counted cells contained detectable U_L_26 expression. (**D**) U_L_26 tegument protein composition of WT and #185stop virus. Viral particles from the supernatants of MRC5 fibroblasts infected with either WT or #185stop virus were separated from cell debris via low-speed centrifugation. Thereafter, the particles were purified by differential sedimentation in a glycerol-tartrate gradient. Resulting virion fractions were processed for Western blotting using U_L_26 and pp65-specific monoclonal antibodies. (**E**) Quantification of tegument U_L_26 protein in glycerol-tartrate purified virions. The amount of U_L_26 protein present in virions (**D**) was quantified after normalization to the amount of virion pp65. Quantification of U_L_26 and pp65 specific bands was performed using BioRad ImageLab software.

Previously, we found that the U_L_26 protein localized to the nucleus at early time points, and moved to the cytoplasm at later time points [23]. To determine whether specific domains of the U_L_26 protein were important for U_L_26 localization during infection, we analyzed cells infected with our panel of U_L_26 mutant viruses using confocal microscopy. After 5 hpi, a time at which the U_L_26 protein should be predominantly tegument-delivered, the U_L_26 protein was localized in the nucleus during WT, and 2^nd^metΔ infection (Fig. 7A). At the same time point, and with equivalent confocal settings, there was reduced staining for nuclear U_L_26 protein in cells infected with the 1stmetΔ (Fig. 7A). U_L_26 protein was not detectable during infection with the #185stop or the U_L_26-null viruses (Fig. 7A). After 72 hpi, the U_L_26 protein exhibits both nuclear and cytoplasmic staining during WT infection (Fig. 7B). After 72 h of infection with the 1^st^metΔ and 2^nd^metΔ viruses, the U_L_26 protein remained primarily nuclear, although there was increased cytoplasmic staining compared to the 5 hpi time point (Fig. 7B). Interestingly, after 72 h of infection with the #185stop virus, U_L_26 was predominantly cytoplasmic, with a reduction in nuclear staining compared to WT or the 1^st^ and 2^nd^metΔ viruses (Fig. 7B). Analysis of multiple fields at this time point revealed that #185stop-U_L_26-protein was capable of accumulating in the nucleus, however it did so in a minority of cells, ∼20%, in comparison to cells infected with wildtype or the methionine mutants, which exhibited nuclear U_L_26 in ∼90% of cells (Fig. 7C). These results indicate that the #185stop mutant is defective for nuclear accumulation, suggesting that the C-terminal 38 amino acids of U_L_26 are important for proper nuclear localization.

As U_L_26 is a tegument protein, and its *de novo* expression depends on the expression of immediate early genes [34], the majority of U_L_26 protein present during the first 5 h of infection is part of the virion that was delivered to the cells upon infection [25]. At 5 h post infection with #185stop, we observed substantially decreased levels of truncated U_L_26, both by western and by immunofluorescence (Fig. 3A & Fig. 7A). These decreased levels of U_L_26 protein could result from defective incorporation into the viral tegument. To explore this possibility, we gradient purified wildtype and #185stop virions. As shown in Figure 7D, #185stop virions contained reduced levels of U_L_26 protein compared to WT virions. There was a greater than 50% reduction in the short #185stop isoform in comparison to wildtype HCMV and a greater than 80% reduction in the long #185stop isoform (Fig. 7E). These results suggest that this C-terminal deletion decreases the tegumentation efficiency of U_L_26 and is likely in part responsible for the reduced abundance of U_L_26 protein at early time points of infection. These results coupled with the defective U_L_26 nuclear localization associated with this mutant suggest that deletion of the C-terminal 38 amino acids largely ablates the nuclear activities associated with U_L_26 at early times post infection.

## Discussion

Herpesvirus tegument proteins play important and divergent roles during the viral life cycle. These proteins are among the first to interact with the host cell upon infection, as they are delivered to the cytoplasm after membrane fusion. At this time, they serve to institute an environment conducive to viral replication, performing a myriad of activities such as suppressing innate immunity, activating cell signaling pathways and inducing viral gene expression [17], [20], [21]. In addition to these early functions, tegument proteins act at the very end of infection, playing roles in the assembly, envelopment, and egress of viral particles [14], [15]. We have found that the U_L_26 tegument protein is critical for high titer HCMV replication [23]. However, it was unclear how the long and short U_L_26 isoforms contribute to *in vitro* HCMV replication. The U_L_26 protein has been implicated in functioning at both early time points of infection, impacting immediate early gene accumulation [23], [25], as well as at late time points during virion assembly, inasmuch as virions lacking U_L_26 exhibit reduced stability [24]. However, how the different U_L_26 isoforms, or how specific U_L_26 domains contribute to these phenotypes is unclear. To address these questions we created a panel of recombinant HCMV U_L_26 mutants and assessed their contribution to HCMV infection. We found that the short U_L_26 isoform is largely dispensable for *in vitro* replication, whereas the N-terminal 34 amino acids of the long isoform are required for wildtype HCMV replication. Further, we find that the C-terminal 38-amino acids of the U_L_26 protein are important for wildtype HCMV replication, as well as for proper nuclear localization and normal tegumentation of the U_L_26 protein.

**Figure 8:**
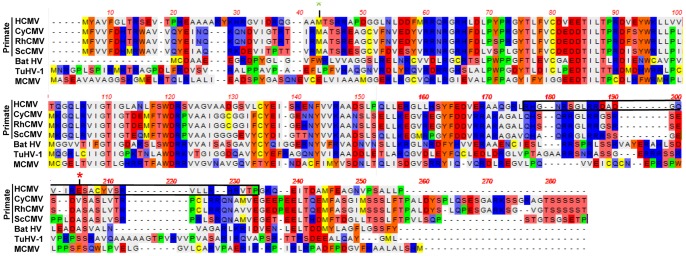
Comparison of U_L_26 protein sequences of various species-specific cytomegaloviruses. The U_L_26 open reading frames of the indicated CMV species were aligned using the NCBI BLAST alignment tool. The black box highlights a putative NLS as predicted by the NLS mapper. The green asterisk indicates the 2^nd^ initiation methionine and the red asterisk indicates the #185-stop insertion site.

The C-terminal 38 amino acids of U_L_26 are critical for U_L_26-protein function, as a truncation mutant lacking these amino acids was indistinguishable from U_L_26-null viruses with respect to IE1 accumulation, viral growth, plaque size, and virion stability. This C-terminal-truncated U_L_26 protein was less abundant at earlier times post infection, but accumulated to wildtype levels at later times post infection. This decrease in U_L_26 protein at early times reflects the decreased tegumentation observed in #185stop virions. This C-terminal truncated U_L_26 protein also displayed substantially reduced nuclear localization compared to wildtype U_L_26. Employing an algorithm for identification of nuclear localization sequences [35], we find that the U_L_26 protein contains a predicted weak nuclear localization signal close to the C-terminus (Fig. 8). The #185stop mutation falls within this sequence, and its deletion is therefore potentially responsible for the defective nuclear localization of this mutant allele (Fig. 8). Given that the #185stop mutant behaves similarly to U_L_26-null virus, and exhibits apparent early and late defects, it makes it difficult to definitively separate early functions from late functions with respect to their contribution to viral replication. Additional site-specific mutational analysis of this region may enable separation of the residues that are important for nuclear localization versus those important for efficient tegumentation. Given its importance for U_L_26 function, mutants of this C-terminal domain will be a powerful tool for further genetic and mechanistic studies into U_L_26’s contribution to HCMV replication. For example, in screening potentially important U_L_26 interacting partners, viral or host-cell factors whose binding is dependent on this C-terminal domain should be given preference with respect to experimental examination.

Interestingly, the C-terminal 38 amino acids of the U_L_26 protein appear to be less well conserved between human, the other primate CMVs, and non-primate CMVs in comparison to other areas of the protein (Fig. 8). Two macaque CMVs, RHCMV and CyCMV, for example, contain an extra ∼30 C-terminal amino acids. This increased divergence between the different U_L_26 proteins may indicate that this domain is important for species specific differences between the CMV strains.

The U_L_26 message contains two initiating methionines which result in two in-frame U_L_26 protein isoforms that differ by only 34 N-terminal amino acids. It is unclear how these different isoforms contribute to HCMV infection. Our results indicate that the shorter isoform is dispensable for many of the *in vitro* phenotypes we assessed, including replication, plaque size, and viral stability. Despite its apparent lack of importance *in vitro*, the second in frame methionine is well conserved in primate CMV species (Figure 8). This suggests that the smaller isoform may be important in other settings, e.g. during *in vivo* infection or infection of alternate cell types. The shorter U_L_26 isoform has been found to be preferentially packaged relative to the longer isoform during tegumentation (Fig. 7C and [25]), however the mechanism governing this difference is not clear. Further, while a putative NLS was predicted in the C-terminus, and should therefore be present in both isoforms, it is not currently clear whether the different isoforms co-localize over the course of viral infection.

While the shorter U_L_26 protein isoform is dispensable for *in vitro* growth, the longer U_L_26 isoform, which contains an additional 34 N-terminal amino acids, was found to contribute to a number of *in vitro* phenotypes. The 1stmetΔ virus exhibited intermediate phenotypes between wildtype and U_L_26-null viruses, including viral growth, plaque size, and viral stability. Further, the 1stmetΔ mutant displayed an MOI-dependent decrease in IE1 accumulation at 4 h. These results indicate that the 34 N-terminal amino acids are important for viral growth and argue that the long and short isoforms are not functionally redundant *in vitro*.

A number of questions remain about U_L_26 and the mechanisms through which it contributes to HCMV replication. Prominently, how does the U_L_26 protein contribute to virion stability? As a tegument protein, physical interactions between the U_L_26 protein and other viral proteins in the virion could be important for maintaining virion stability. Alternatively, U_L_26’s contribution to stability may occur earlier, for example in the proper assembly of virion particles. Previously we found that U_L_26-null viruses produce virions with hypophosphorylated tegument proteins [23]. It is unclear whether this hypophosphorylation could contribute to unstable virions, or alternatively, whether the hypophosphorylation is a consequence of virion destabilization. Analysis of purified virion proteins by silver-stained gel indicates there is no dramatic difference in the proteins present in wildtype versus U_L_26-null virus (data not shown). This suggests that large differences in virion protein constituents are likely not responsible for the decreased stability of mutant U_L_26 viruses. Another major question is the function of U_L_26 in the nucleus at early times. It seems likely that this nuclear U_L_26 is responsible for impacting IE1 gene expression at early times, although the potential mechanism involved still needs to be elucidated. Our identification of the important U_L_26 N-terminal and C-terminal domains will facilitate addressing these questions. The C-terminal 38-amino acids of U_L_26 are important for proper U_L_26 tegumentation, nuclear localization, and viral replication. Our studies highlight the importance of these C-terminal 38-amino acids for future study. Further functional analysis will distinguish how the specific residues within this domain contribute to U_L_26 nuclear localization and proper tegumentation, and subsequently to HCMV replication. Given its importance to HCMV infection, elucidating the mechanisms through which U_L_26 domains contribute to high-titer replication may shed light on possibilities for therapeutic intervention.
