# Correction: Distinct Domains within the Human Cytomegalovirus U_L_26 Protein Are Important for Wildtype Viral Replication and Virion Stability

**DOI:** 10.1371/journal.pone.0114927

**Published:** 2014-12-01

**Authors:** The PLOS ONE Staff

Figure 5 was incorrectly published as a duplicate of Figure 7. Please see the corrected Figure 5 here.

**Figure 5:**
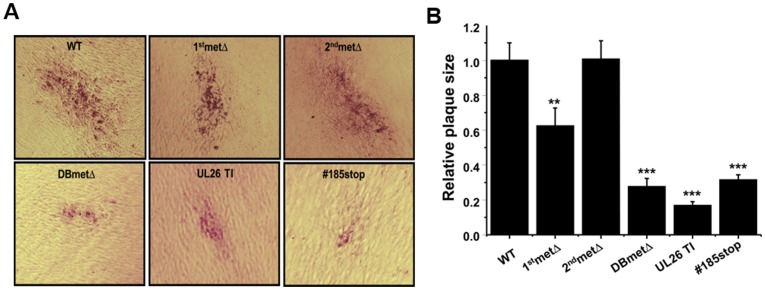
Analysis of HCMV plaque formation. (**A**) Replicate cultures of MRC5 fibroblasts were infected with 25 PFU of the indicated recombinant virus. Representative plaques at day 15 post infection for each virus are shown. (**B**) Areas of representative plaques for each virus were quantified by Image J and normalized to the WT plaque size. Values are means+SE (n  =  10). **  =  p<0.01; ***  =  p<0.001.

## References

[1] Mathers C, Spencer CM, Munger J (2014) Distinct Domains within the Human Cytomegalovirus U_L_26 Protein Are Important for Wildtype Viral Replication and Virion Stability. PLoS ONE 9(2): e88101 doi:10.1371/journal.pone.0088101 24505393 10.1371/journal.pone.0088101 PMC3914908

